# Soybean Oil in Propofol Is Not Able to Induce Allergic Reactions

**DOI:** 10.1111/all.16454

**Published:** 2024-12-29

**Authors:** Deniz Eyice Karabacak, Lisa Arzt‐Gradwohl, Barbara Binder, Eva Schadelbauer, Christina Vallant, Karin Laipold, Gunter J. Sturm

**Affiliations:** ^1^ Division of Immunology and Allergic Diseases, Department of Internal Medicine, Faculty of Medicine Istanbul University Istanbul Türkiye; ^2^ Department of Dermatology and Venereology Medical University of Graz Graz Austria; ^3^ Allergy Outpatient Clinic Reumannplatz Vienna Austria

**Keywords:** anesthesia, birch pollen allergy, propofol, skin prick test, soy, soybean oil, soymilk

To the Editor,

The use of propofol in patients allergic to soy and peanut is still a debated issue since it contains soybean oil [1, 2]. In previous studies, sensitization to soy and peanut detected by skin test and specific IgE (sIgE) determination was not associated with allergic reactions during anesthesia with propofol [3, 4]. However, according to the summary of product characteristics (SmPC), using propofol is still contraindicated in patients with hypersensitivity to soy and peanut. These precautionary measures are not justified because refined soybean oil contains insignificant amounts of protein. Furthermore, if cross‐sensitizations to soy and peanut due to birch pollen allergy were clinically relevant, a higher prevalence would be expected. In this study, we aimed to evaluate if soybean oil is able to induce positive skin prick test (SPT) reactions in a large number of subjects who underwent allergy diagnosis. Between January 2016 and December 2021, soymilk and soybean oil were added to the routine SPT panel of the Allergy Outpatient Clinic Reumannplatz in Vienna and the Allergy Outpatient Clinic of the Department of Dermatology and Venereology of the Medical University of Graz. Routine SPTs were performed with extracts from ALK‐Abelló (Hørsholm, Denmark), the prick‐to‐prick test was done with soymilk and soybean oil. Results were considered positive in cases of a wheal larger than 3 mm in diameter and erythema. Specific IgE levels were detected using the ImmunoCAP 1000 (Thermo Fisher Scientific, Waltham, USA); values > 0.35 kU/L were considered positive.

In total, 17,540 consecutive patients were included in this retrospective analysis (see Figure 1). Of these, 2967 patients had a positive SPT to birch pollen. In 107 patients, a sensitization to soy or soymilk was detected: 20 patients had positive sIgE levels to soy extract, while 87 patients had a positive SPT to soymilk. Importantly, the SPT with soybean oil was negative in all patients. In addition, 79 patients were sensitized to peanut (26 patients had positive sIgE levels and 53 patients had a positive SPT).

**FIGURE 1:**
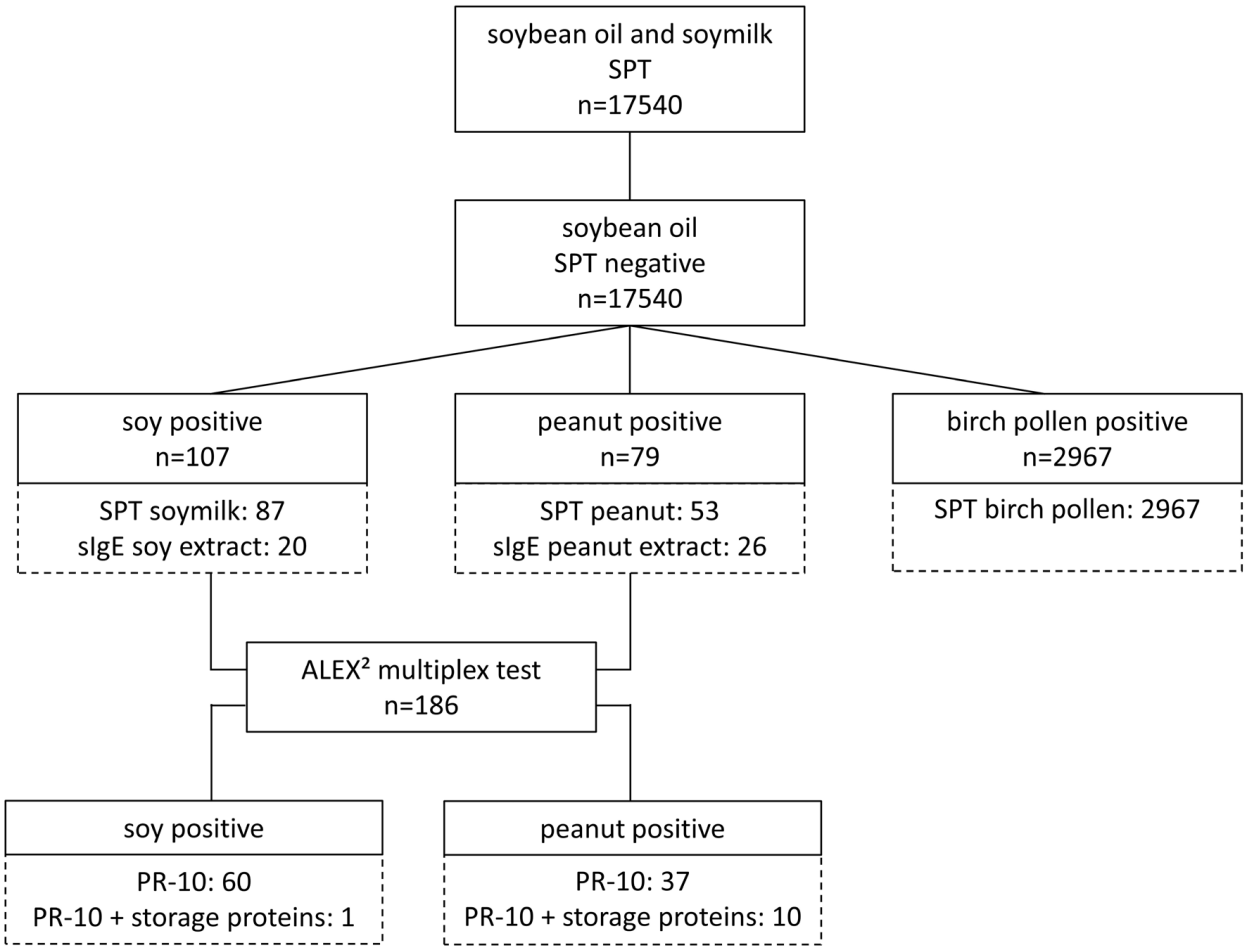
Flowchart of study results. sIgE, specific immunoglobulin E; SPT, skin prick test.

For all patients with a sensitization either to soy or peanut (*n* = 186), the multiplex test ALEX^2^ (MacroArray Diagnostics, Vienna, Austria) was performed; values > 0.30 kU_A_/L were considered positive.

Of the 107 patients sensitized to soy or soymilk, 60 (56.1%) had a PR‐10 (Gly m 4 and Ara h 8) sensitization, one of them was also positive to peanut storage proteins. Fifty‐nine of the PR‐10 sensitized patients had positive SPT results to soymilk. Four (3.7%) patients had a reaction to soy in the past: three had a systemic reaction and one patient had an oral allergy syndrome (OAS). In the group of patients with peanut sensitization, 37 (46.8%) had a PR‐10 (Gly m 4 and Ara h 8) sensitization, ten of them were also positive to peanut storage proteins. Thirty of them had positive SPT results to peanut. A total of 16 patients reacted to peanut: three patients had a systemic reaction while 13 suffered from an OAS. In the remaining patients, no sensitization to available molecular allergens could be detected.

To conclude, we could demonstrate that soybean oil was not able to induce positive skin tests in a large number of patients irrespective of whether they had a confirmed birch pollen allergy, a genuine sensitization to soy or peanut, a sensitization to cross‐reactive allergens (Gly m 4 and Ara h 8) or no sensitization at all. Possible limitations of the study include the small number of patients with systemic allergies to peanut and soy and the fact that prick‐to‐prick testing is not a standardized method. However, our study adds further evidence to the existing literature that the risk of allergic reactions to soybean oil in propofol is neglectable. It would be important to adapt the SmPC to avoid unnecessary allergological work‐up, or even worse, the use of inferior, alternative drugs.

## Author Contributions

Conceptualization: Deniz Eyice Karabacak and Gunter J. Sturm. Supervision: Gunter J. Sturm. Project administration: Lisa Arzt‐Gradwohl. Investigation: Deniz Eyice Karabacak, Lisa Arzt‐Gradwohl, Barbara Binder, Eva Schadelbauer, Christina Vallant and Gunter J. Sturm. Formal analysis: Deniz Eyice Karabacak, Gunter J. Sturm and Lisa Arzt‐Gradwohl. Methodology: Deniz Eyice Karabacak and Karin Laipold. Writing – original draft: Deniz Eyice Karabacak, Lisa Arzt‐Gradwohl and Gunter J. Sturm. Writing – review and editing: Deniz Eyice Karabacak, Lisa Arzt‐Gradwohl, Barbara Binder, Eva Schadelbauer, Christina Vallant, Karin Laipold and Gunter J. Sturm. The corresponding author attests that all listed authors meet authorship criteria and that no others meeting the criteria have been omitted.

## Conflicts of Interest

Dr. Deniz Eyice Karabacak has nothing to disclose. Dr. Lisa Arzt‐Gradwohl has nothing to disclose. Dr. Barbara Binder has nothing to disclose. Dr. Eva Schadelbauer has nothing to disclose. Dr. Christina Vallant has nothing to disclose. Ms. Karin Laipold has nothing to disclose. Dr. Gunter J. Sturm reports grants from ALK‐Abelló, personal fees from ALK‐Abelló, personal fees from Allergopharma, personal fees from Novartis, and personal fees from Stallergenes‐Greer, outside the submitted work.

## Data Availability

The data that support the findings of this study are available from the corresponding author upon reasonable request.
